# The Impact of Fertilizer Amendments on Soil Autotrophic Bacteria and Carbon Emissions in Maize Field on the Semiarid Loess Plateau

**DOI:** 10.3389/fmicb.2021.664120

**Published:** 2021-06-18

**Authors:** Jinbin Wang, Junhong Xie, Lingling Li, Zhuzhu Luo, Renzhi Zhang, Linlin Wang, Yuji Jiang

**Affiliations:** ^1^Gansu Provincial Key Laboratory of Aridland Crop Science, Lanzhou, China; ^2^College of Agronomy, Gansu Agricultural University, Lanzhou, China; ^3^College of Resource and Environment, Gansu Agricultural University, Lanzhou, China; ^4^State Key Laboratory of Soil and Sustainable Agriculture, Institute of Soil Science, Chinese Academy of Sciences, Nanjing, China

**Keywords:** fertilization amendment, soil autotrophic bacteria, carbon emission efficiency, crop yield, semiarid Loess Plateau

## Abstract

Soil autotrophic bacteria play a crucial role in regulating CO_2_ fixation and crop productivity. However, the information is limited to how fertilization amendments alter soil autotrophic bacterial community, crop yield, and carbon emission efficiency (CEE). Here, we estimated the impact of the structure and co-occurrence network of soil autotrophic bacterial community on maize yield and CEE. A long-term field experiment was conducted with five fertilization treatments in semiarid Loess Plateau, including no amendment (NA), chemical fertilizer (CF), chemical fertilizer plus commercial organic fertilizer (SC), commercial organic fertilizer (SM), and maize straw (MS). The results showed that fertilization amendments impacted the structure and network of soil Calvin–Benson–Bassham (CBB) (*cbbL*) gene-carrying bacterial community via changing soil pH and NO_3_–N. Compared with no amendment, the *cbbL*-carrying bacterial diversity was increased under the SC, SM, and MS treatments but decreased under the CF treatment. Soil autotrophic bacterial network contained distinct microbial modules that consisted of closely associated microbial species. We detected the higher abundances of soil *cbbL*-carrying bacterial genus *Xanthobacter*, *Bradyrhizobium*, and *Nitrosospira*. Structural equation modeling further suggested that the diversity, composition, and network of autotrophic bacterial community had strongly positive relationships with CEE and maize yield. Taken together, our results suggest that soil autotrophic bacterial community may drive crop productivity and CEE, and mitigate the atmospheric greenhouse effect.

## Introduction

The growing emissions of greenhouse gasses, carbon dioxide (CO_2_), nitrous oxide (N_2_O), and methane (CH_4_), contributing to global warming are of great concern worldwide (Mora et al., 2018). Soil respiration is the second largest carbon flux between the terrestrial ecosystem and the atmosphere. Carbon dioxide released into the atmosphere through soil respiration has a significant effect on the global C budget (Peylin et al., 2013) and global warming (Cherubini et al., 2011). Annually, CO_2_ emission from soil accounts for 25% of the global total C exchange between the atmosphere and terrestrial sources (Schlesinger and Andrews, 2000), and agroecosystems represent a significant source of CO_2_ emission. Agronomic management practices such as tillage and fertilization have a marked effect on soil ecosystem and soil CO_2_ emission (Ding et al., 2007; Dong et al., 2009). Plants assimilate atmospheric CO_2_ through photosynthesis, increase carbon use efficiency, and reduce the atmospheric CO_2_ concentration (Eric et al., 2018). Therefore, reducing CO_2_ emissions and increasing carbon assimilation under agricultural practices are effective strategies for mitigating atmospheric greenhouse effect in the context of global climate change.

Soil microorganisms play key roles in mediating soil organic matter and nutrient cycling dynamics and profoundly impact soil productivity and sustainability (Theuerl and Buscot, 2010; Nimmo et al., 2013). Soil autotrophic bacteria play an essential role in soil C fixation through the Calvin–Benson–Bassham (CBB) cycle to utilize atmosphere CO_2_ (Selesi et al., 2005). Ribulose-1,5-bisphosphate carboxylase/oxygenase (RubisCO) is responsible for catalyzing the first rate-limiting step in the CBB cycle for autotrophic CO_2_ fixation. To date, four RubisCO forms (forms I–IV) have been found that differ in structure, catalytic property, and O_2_ sensitivity. Form I of RubisCO is the most abundant among the four forms. The *cbbL* gene, which encodes a large subunit of RubisCO I, has been often used as a phylogenetic marker to investigate the autotrophic bacterial community (Kovaleva et al., 2011). Previous studies have reported that fertilization treatments significantly affect the *cbbL*-carrying bacterial community structure and, consequently, facilitate carbon sequestration (Yuan et al., 2012b). So far, the question remains as to whether and how fertilization treatments alter the structure and network of the soil *cbbL*-carrying bacterial community.

Fertilization treatments can significantly affect nutrient-supplying (Yousaf et al., 2017), soil quality, and grain yield (Steiner et al., 2007; Alburquerque et al., 2013). Long-term reliance on chemical fertilizers leads to a decline in soil quality (Liu et al., 2010), nutrient imbalance (Mandal et al., 2007), and reduced fertilizer use efficiency (Duan et al., 2014). Organic fertilizer plus chemical fertilizers can increase soil organic C and enhance soil fertility (Mandal et al., 2007; Diacono and Montemurro, 2011). Furthermore, fertilization enhances CO_2_ fixation, carbon emission efficiency (CEE), and crop yield via promoting soil autotrophic bacterial activity (Selesi et al., 2005; Yuan et al., 2012b; Souza et al., 2015). However, information on the direction and extent of autotrophic bacteria that govern soil CEE and crop yield under different fertilization treatments is limited.

On the Chinese semiarid Loess Plateau, maize is an important crop, where low rainfall (Xin and Lu, 2011) and low heat units (Gan et al., 2013) challenge crop yield. The fully plastic mulched ridge-furrow system has become a widely adopted practice for producing high crop yields and water use efficiency in recent years (Rong et al., 2013).

The intent of our study was to investigate the impact of the structure and network of soil autotrophic bacterial community on CEE and maize yield. We performed a long-term field experiment with five fertilization treatments on the semiarid Loess Plateau. Specifically, the objectives of this study were to (i) investigate the diversity, composition, and co-occurrence network of soil autotrophic bacterial community under fertilization treatments; and (ii) explore the underlying mechanisms of soil properties and the autotrophic bacterial community in regulating C emission, CEE, and maize yield. We hypothesized that organic manure plus chemical fertilizers increased soil autotrophic bacterial diversity and changed composition and network of soil autotrophic bacterial community. Furthermore, we expected that soil properties and autotrophic bacterial community jointly regulated carbon emission, CEE, and crop yield.

## Materials and Methods

### Field Experiment Description

The long-term fertilization experiment commenced at the Rainfed Agricultural Experimental Station (35°28′N, 104°44′E, elevation 1,971 m above sea level) of Gansu Agricultural University in Gansu province, China. The experimental site is at the temperature semiarid zone in the western Loess Plateau, with a long-term average solar radiation of 593 kJ cm^–2^ and an annual average sunshine of 2,477 h. The mean air temperature is 6.4°C, with accumulated temperature > 0°C of 2,934°C and > 10°C of 2,239°C. The aeolian soil at the experimental site is locally known as Huangmian (Gong, 2001), which is a Calcaric Cambisol according to the Food and Agriculture Organization (FAO) soil classification (FAO, 1990).

The long-term field experiment followed a completely randomized design with three replications. The experiment was conducted in 2012, which consist of 15 plots with the following treatments: no amendment (NA) and four fertilization treatments with the same nitrogen (N) (200 kg N ha^–1^) and phosphate (150 kg P_2_O_5_ ha^–1^) input: (1) chemical fertilizer (CF), applied 200 kg N ha^–1^ as urea (46% N) plus 150 kg P_2_O_5_ ha^–1^ triple superphosphate (16% P_2_O_5_); (2) 50% chemical N fertilizer plus 50% organic N fertilizer (SC), applied 3.03 t ha^–1^ of commercial organic fertilizer plus 100 kg N ha^–1^ as urea and 120 kg P_2_O_5_ ha^–1^ as triple superphosphate; (3) organic fertilizer (SM), applied 6.06 t ha^–1^ of commercial organic fertilizer plus 90 kg P_2_O_5_ ha^–1^ as triple superphosphate; and (4) maize straw (MS), applied at 28.5 t ha^–1^ plus 36 kg ha^–1^ as triple superphosphate. The commercial organic fertilizer used contained 3.3% N, 1.0% P_2_O_5_, and 0.7% K_2_O, with cow manure as ripened organic fertilizer (Gansu Daxing Agricultural Technology Co., Gansu, China). The maize straw obtained from the previous harvesting season was air-dried, shredded to ≤ 5 cm, weighed, and then applied to the field plots. The maize straw contained 0.7% N, 0.4% P_2_O_5_, and 0.5% K_2_O. Representative samples of maize straw were collected at the time of application and analyzed for nutrient concentration using an Elementar Vario MACRO Cube (Elementar, Hanau, Germany). All fertilizers were evenly applied on the soil surface and incorporated by moldboard plowing to a depth of 25 cm in the spring.

The experimental plots were 42.9 m^2^ (13 m long and 3.3 m wide) and consisted of narrow ridges (15 cm high × 40 cm wide) alternated with wide ridges (10 cm high × 70 cm wide) (Supplementary Figure 1). Plastic film (0.01 mm thick and 140 cm wide; Lanzhou Green Garden Corp., Lanzhou, China) covered all ridges to increase soil temperature and reduce evaporative losses. After the plastic film is placed, a handheld device made holes through the film’s furrows to channel rainfall from the ridges into the root zone. Maize (cv. Pioneer 335) was sown in late April and harvested in mid-October with a plant spacing in each row at 35 cm with a density of 52,500 plants ha^–1^. Weeds were manually removed during the maize growing season. All agronomic practices, except for the fertilization amendments, were kept uniform in this study.

### Soil Sampling and Soil Property Assays

At silking stage of maize in early August 2019, five randomly selected soil cores (5 cm in diameter) to 20-cm depth from each plot were passed through a 2-mm sieve to remove residues and mixed as one composite sample. A subsample from each composite sample was immediately placed with dry ice for transport to the laboratory and stored at –80°C for DNA analysis, while the remaining soil was air-dried for soil chemical analysis.

Soil pH was measured in distilled water (soil:water ratio of 1:2.5 m/v) using a pH meter (Mettler Toledo FE20, Shanghai, China). A modified Walkley–Black wet oxidation method and the Kjeldahl method were used to determine soil organic carbon (SOC) and total nitrogen (TN), respectively (Bao, 2000). Soil NO_3_–N and NH_4_–N were extracted with 2 M of KCl and measured using a continuous flow analyzer (Skalar, Breda, Netherlands). Available phosphorus (AP) was extracted with sodium bicarbonate and measured using the molybdenum-blue method (Olsen, 1954).

### Soil DNA Extraction and Amplification

Total genome DNA from soil samples was extracted using an OMEGA soil DNA Kit (Omega Bio-Tek, Doraville, GA, United States). The quantity and quality of DNA were determined by agarose gel (1%) electrophoresis and a NanoDrop 2000 (Thermo Fisher Scientific, Waltham, MA, United States). According to the concentration, DNA was diluted to 1 ng μl^–1^ using sterile water. The specific primer K2f/V2r, ACCAYCAAGCCSAAGCTSGG/GCCTTCSAGCTTGCCSACCRC was used to amplify the autotrophic bacterial community (Tolli and King, 2005). All PCR were carried out in 30-μl reactions with 15 μl of PCR Master Mix (New England Biolabs, Ipswich, MA, United States), 0.2 μM of forward and reverse primers, and 10 ng of template DNA. Thermal cycling consisted of initial denaturation at 98°C for 1 min, followed by 30 cycles of denaturation at 98°C for 10 s, annealing at 50°C for 30 s, and elongation at 72°C for 60 s. The PCR products remained at 72°C for 5 min, and then the same volume of 1 × loading buffer was mixed with the PCR products and analyzed by electrophoresis on 2% agarose gel. The PCR products were then mixed in equivalent ratios and purified with a GeneJET Gel Extraction Kit (Thermo Fisher Scientific, Waltham, MA, United States).

### Illumina MiSeq Sequencing

The library was prepared using the NEB Next Ultra II DNA library prep kit for Illumina (New England Biolabs, Ipswich, MA, United States) according to manufacturer’s instructions. The library quality was assessed using a Qubit@ 2.0 Fluorometer (Thermo Fisher Scientific, Ipswich, MA, United States) and the Agilent Bioanalyzer 2100 system (Agilent Tec., Palo Alto, CA, United States). The library was sequenced on an Illumina MiSeq platform (Shanghai Biozeron Biotechnology Co., Ltd, Pudong, Shanghai, China), generating 250-bp paired-end reads. Paired-end reads from the original bacterial DNA fragments were merged using FLASH (version 1.2.7)^1^. Sequence analyses were performed with the UPARSE (version 7.1)^2^ software package using the UPARSE-OTU and UPARSE-OTUref algorithms (Edgar, 2013), and the operational taxonomic unit (OTU) was clustered at a distance of ≤ 0.03 (approximately 97% of the sequence similarity). Alpha-diversity of the autotrophic bacterial community was calculated after rarefying all samples to an equal number of 24,138 reads. Alpha-diversity analysis was performed using MOTHUR software (Schloss et al., 2009), and diversity indices were determined, including the Chao 1, Shannon, and Simpson indices (Caporaso et al., 2010). We have deposited sequencing reads in the Sequence Read Archive at National Center for Biotechnology Information (NCBI) under the accession number PRJNA700109.

### Soil Respiration, Total Carbon Emission, Grain Yield, and Carbon Emission Efficiency

In order to minimize the root effect, the plastic tube was pressed in the big ridge with a distance of 25 cm far from maize plants. Polyvinyl chloride collars that were 20 cm in diameter and 11.5 cm in height were pressed into the soil post-crop emergence to a depth of 9.5 cm, leaving 2 cm exposed above the soil surface and under the plastic mulch. These collars were used to locate the LI-8100 soil chamber (LI-COR Inc., Lincoln, NE, United States) and provided an airtight seal. Soil respiration was measured at 09:00, 11 times per year from May 3 to October 16 in 2018 and from May 5 to October 17 in 2019. Twelve hours before the soil measurements, the plastic mulch was removed from the collars to release emissions under the mulch. After the sampling, the plastic mulch was replaced.

Carbon emission (kg ha^–1^) was calculated based on soil respiration (μmol m^–2^ s^–1^) using the following equation (Zhai et al., 2011):

(1) $$CE=\sum [\frac{R\,s\,\left(i+1\right)+R\,s\,\left(i\right)}{2}\times \left[t\left(i+1\right)-t\left(i\right)\right].\times 0.1584\times 24]\times 0.2727\times 10$$

where Rs is soil respiration (μmol CO_2_ m^–2^ s^–1^) measured at 15-day intervals during the growing season; i + 1 and i are the current and previous sampling dates, respectively; t is days after sowing; 0.1584 converts μmol CO_2_ m^–2^ s^–1^ to g CO_2_ m^–2^ h^–1^; 0.2727 converts g CO_2_ m^–2^ h^–1^ to g C m^–2^ h^–1^; and 24 and 10 convert g C m^–2^ h^–1^ to kg ha^–1^ for the growing season.

Plots were harvested at physiological maturity of maize, and yield was determined on an oven-dry-weight basis by drying the grain at 105°C for 45 min and subsequently to constant weight at 85°C (Lamptey et al., 2017).

CEE (kg kg^–1^) was calculated from crop yield per unit of C emission using the following equation (Qin et al., 2013):

(2) $$C\,E\,E=\frac{C\,r\,o\,p\,y\,i\,e\,l\,d}{c\,a\,r\,b\,o\,n\,e\,m\,i\,s\,s\,i\,o\,n}$$

### Data Analysis

Analysis of variance was performed by Fisher’s protected least significant (*P* < 0.05) difference test using SPSS software (v. 20.0, IBM Corp., Chicago, IL, United States). Pearson’s correlation coefficients were used to assess linear associations among soil properties, communities of soil *cbbL*-carrying bacteria, crop yield, total carbon emission (TCE), and CEE across soil amendment. All statistical analyses were conducted based on 15 samples (5 treatments × 3 replicates). Principal coordinate analysis (PCoA) was used to evaluate the Bray–Curtis distances of soil *cbbL*-carrying bacterial community compositions using the “vegan” package in R (version 4.0.2) software.

To describe the complex co-occurrence patterns in soil *cbbL*-carrying bacterial networks, co-occurrence networks were constructed using Spearman’s correlation and the Kullback–Leibler dissimilarity (KLD) measures (Faust and Raes, 2012). The OTUs detected in more than two-thirds of the soil samples were kept for network construction. A valid co-occurrence was considered a statistically robust correlation between species when the correlation coefficient (*r*) was > 0.6 or less than -0.6 and the *P* value was < 0.05. The co-occurrence network visualization was conducted using Gephi software (Bastian et al., 2009), and modules were defined as clusters of closely interconnected nodes (i.e., groups of co-occurring microbes) (Layeghifard et al., 2017).

Random forest tool was used to quantitatively evaluate the important predictors of crop yield and CEE. Random forest modeling was conducted using the RandomForest package (Bento et al., 2002), and the rfPermute package (Archer, 2016) was used to determine the model significance and predictor importance. The significant predictors were further chosen to perform a structural equation modeling (SEM) analysis. SEM analysis was applied to determine the contributions of soil properties and soil *cbbL*-carrying bacterial community to the maize yield and CEE. SEM analysis was conducted via the robust maximum likelihood evaluation method using AMOS 22.0 (SPSS, Chicago, IL, United States). Latent variables were used to integrate the effects of multiple conceptually related observed variables into a single-composite effect, aiding interpretation of model results. The SEM fitness was examined on the basis of a nonsignificant chi-square test (*P* > 0.05), the goodness-of-fit index, and the root mean square error of approximation (Hooper et al., 2008).

## Results

### Soil Properties, C Emission, Crop Yield, and Carbon Emission Efficiency

Soil chemical properties were significantly affected by soil amendment (*P* < 0.05, Table 1). Soil TN, NO_3_–N, and AP were significantly (*P* < 0.05) increased under four fertilization treatments compared with the NA treatment, while soil pH exhibited a significant decreasing trend (*P* < 0.05). SOC under the SC, SM, and MS treatments was significantly greater than that under NA and CF treatments (*P* < 0.01). However, there was no significant difference in NH_4_–N among the five treatments (*P* = 0.454).

**TABLE 1:** Soil chemical properties at the flowering stage of maize under five treatments.

Treatments	pH	TN (g kg^–1^)	SOC (g kg^–1^)	NO_3_–N (mg kg^–1^)	NH_4_–N (mg kg^–1^)	AP (mg kg^–1^)
NA	8.66 ± 0.03a	0.85 ± 0.01b	7.48 ± 0.18c	17.84 ± 1.04c	15.33 ± 1.63a	9.73 ± 1.41c
CF	8.32 ± 0.07c	0.93 ± 0.03a	7.93 ± 0.07c	30.81 ± 2.78a	16.07 ± 1.48a	16.70 ± 1.57ab
SC	8.44 ± 0.08bc	0.93 ± 0.02a	8.84 ± 0.20b	28.40 ± 3.57ab	14.87 ± 2.22a	18.32 ± 1.69ab
SM	8.45 ± 0.05bc	0.98 ± 0.03a	8.81 ± 0.33b	25.40 ± 3.41abc	15.81 ± 1.36a	19.81 ± 0.72a
MS	8.54 ± 0.06b	0.99 ± 0.002a	9.82 ± 0.19a	21.93 ± 1.81bc	16.53 ± 2.80a	15.14 ± 0.97b

*Within a column for a given dependent variable, means ± standard error followed by different letters are significantly different at *P* < 0.05. TN, total nitrogen; SOC, soil organic carbon; NO_3_–N, nitrate nitrogen, NH_4_–N, ammonium nitrogen; AP, available phosphorus; NA, no amendment; CF, chemical fertilizer; SC, 50% chemical N fertilizer plus 50% organic N fertilizer; SM, organic fertilizer; MS, maize straw.*

**FIGURE 1:**
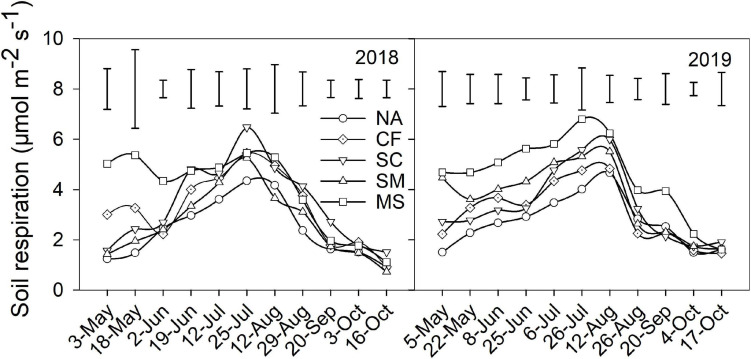
Soil respiration under five treatments during the 2018 and 2019 growing seasons. Vertical bars indicate the least significant difference value at *P* < 0.05. NA, no amendment; CF, chemical fertilizer; SC, 50% chemical N fertilizer plus 50% organic N fertilizer; SM, organic fertilizer; MS, maize straw.

During the maize growing seasons, soil respiration exhibited a similar pattern across five treatments in 2018 and 2019 (Figure 1). Soil respiration gradually increased from sowing in May and reached a maximum in July and then decreased to a minimum at harvest in October. Compared with the NA treatment, the MS treatment significantly increased in mean soil respiration and C emission by 63.6% and 56.7% in 2018 and by 69.5% and 68.8% in 2019, followed by the CF, SC, and SM treatments (Table 2). Crop yield and CEE significantly increased under CF and SC treatments compared with SM, MS, and NA treatments in 2018 and 2019 (*P* < 0.01). However, TCE was not significantly different under CF, SC, and SM treatments (Table 2).

**TABLE 2:** Mean soil respiration (Rs), total carbon emission (TCE), crop yield, and carbon emission efficiency (CEE) in 2018 and 2019 under five treatments.

Year	Treatments	Mean Rs (μmol m^–2^ s^–1^)	TCE (kg ha^–1^)	Yield (kg ha^–1^)	CEE (kg kg^–1^)
2018	NA	2.42 ± 0.14c	4503 ± 282c	4384 ± 247c	0.99 ± 0.12c
	CF	3.25 ± 0.47ab	5937 ± 836ab	11021 ± 512a	1.91 ± 0.18a
	SC	3.42 ± 0.08ab	6390 ± 161ab	9698 ± 801a	1.53 ± 0.17ab
	SM	2.67 ± 0.07bc	4994 ± 123bc	6284 ± 280b	1.26 ± 0.09bc
	MS	3.96 ± 0.17a	7054 ± 339a	5916 ± 478bc	0.84 ± 0.08c
2019	NA	2.72 ± 0.28c	4652 ± 430c	4602 ± 73c	1.01 ± 0.12b
	CF	3.10 ± 0.16bc	5311 ± 260bc	11329 ± 939a	2.14 ± 0.17a
	SC	3.39 ± 0.27bc	5780 ± 546bc	9930 ± 316a	1.75 ± 0.20a
	SM	3.69 ± 0.17b	6209 ± 230b	6205 ± 107b	1.00 ± 0.05b
	MS	4.61 ± 0.17a	7853 ± 340a	5207 ± 273bc	0.68 ± 0.06b

*Within a column for a given dependent variable, means ± standard error followed by different letters are significantly different at *P* < 0.05. NA, no amendment; CF, chemical fertilizer; SC, 50% chemical N fertilizer plus 50% organic N fertilizer; SM, organic fertilizer; MS, maize straw.*

### Community Structure of Soil Autotrophic Bacteria

**FIGURE 2:**
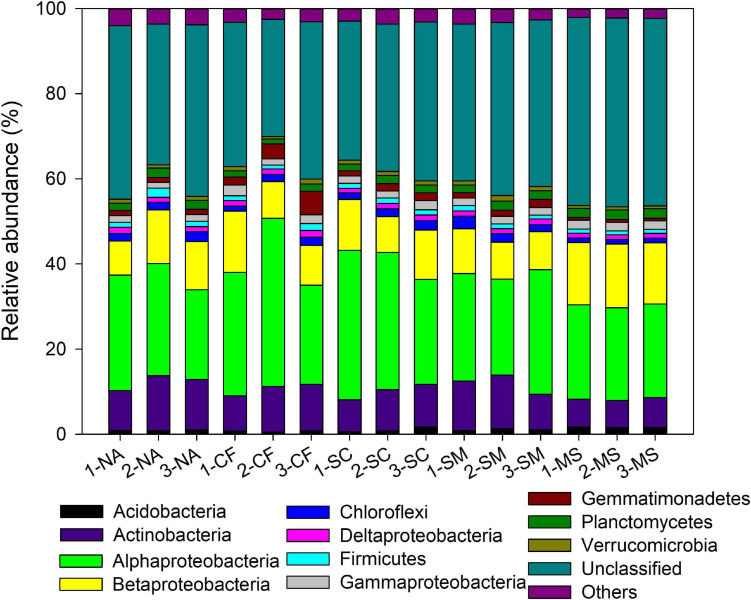
Relative abundance of the autotrophic bacterial phyla under five treatments. NA, no amendment; CF, chemical fertilizer; SC, 50% chemical N fertilizer plus 50% organic N fertilizer; SM, organic fertilizer; MS, maize straw. The numbers before the treatment name indicate the sampling replications. For example, 1-NA, 2-NA, and 3-NA mean the sampling was taken from replicates 1, 2, and 3 of the field plots, respectively.

**FIGURE 3:**
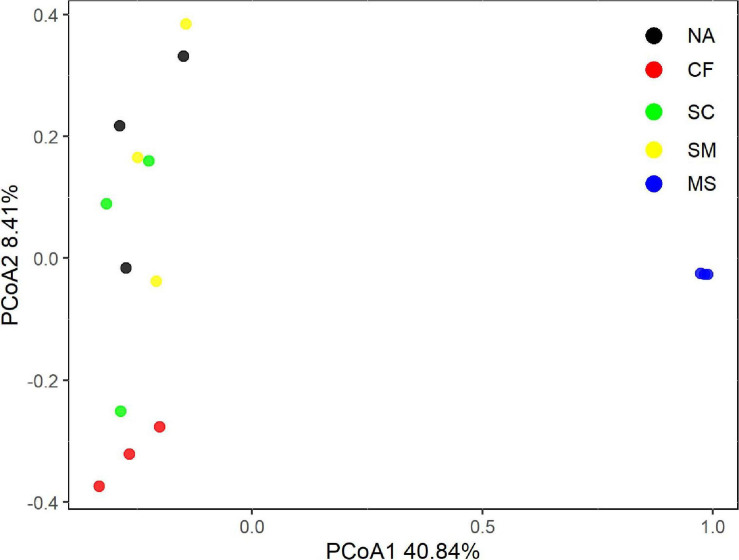
The structure of soil autotrophic bacterial community by principal coordinate analysis (PCoA), which is constrained by five treatments based on Bray–Curtis distances. NA, no amendment; CF, chemical fertilizer; SC, 50% chemical N fertilizer plus 50% organic N fertilizer; SM, organic fertilizer; MS, maize straw.

With the use of Illumina sequencing of *cbbL*-carrying bacteria amplicons, a total of 362,070 sequences were obtained after quality control. The alpha-diversity of the autotrophic bacterial community indicated by Shannon index and Chao 1 richness was significantly (*P* < 0.05) higher under the MS treatment than under the CF, NA, SC, and SM treatments (Supplementary Table 1). Across all samples, the autotrophic bacterial community consisted mainly of Alphaproteobacteria (26.7%), Betaproteobacteria (11.2%), and Actinobacteria (9.6%) (Figure 2). The dominant community closely matched facultative chemoautotrophic hydrogen oxidizers within the genera *Xanthobacter* (11.1%), *Bradyrhizobium* (6.6%), and *Nitrosospira* (6.4%) (Supplementary Figure 2). The results of PCoA, based on the Bray–Curtis distance, showed that soil autotrophic bacterial community structure significantly differed among five treatments (Figure 3). The PCoA1 and PCoA2 explained 40.8% and 8.4%, respectively, of the variation in soil autotrophic bacterial community composition. The composition of soil autotrophic bacterial community under SM and SC treatments was clustered together with the NA treatment, whereas those communities under the CF and MS treatments were clearly separated from the NA treatment. The diversity of soil autotrophic bacteria was significantly correlated with pH (*r* = 0.56, *P* < 0.05), TN (*r* = 0.66, *P* < 0.01), and SOC (*r* = 0.80, *P* < 0.01), while the community composition was significantly correlated with SOC (*r* = 0.74, *P* < 0.01) (Table 3). The diversity and composition of soil autotrophic bacterial community had significantly positive relationships with TCE (*r* = 0.75, *P* < 0.01 and *r* = 0.72, *P* < 0.01) but negative correlations with CEE (*r* = -0.66, *P* < 0.01 and *r* = -0.62, *P* < 0.01) and crop yield (*r* = -0.61, *P* < 0.05 and *r* = -0.53, *P* < 0.05) (Table 3).

**TABLE 3:** Correlation coefficients between soil properties, the diversity and composition of soil autotrophic bacterial community, crop yield, total carbon emission (TCE), and carbon emission efficiency (CEE).

	pH	TN	SOC	NO_3_–N	NH_4_–N	AP	Grain yield	TCE	CEE
Diversity	0.56*	0.66**	0.80**	–0.19	0.44	0.18	−0.61*	0.75**	−0.66*
Composition	0.27	0.51	0.74**	–0.28	0.21	–0.06	−0.53*	0.72**	−0.62*
Module I	–0.26	0.00	–0.36	0.75**	0.3	0.36	0.79**	–0.26	0.95***
Module II	0.15	0.59*	0.64*	0.03	0.42	0.06	–0.12	0.74**	–0.19
Module III	0.35	0.59*	0.78**	–0.11	0.38	0.07	–0.31	0.73**	–0.41

The soil autotrophic bacterial community include diversity (Shannon index), composition [first principal coordinates (PCoA1)], and the eigengenes of three modules (modules I, II, and III) in trophic co-occurrence network. TN, total nitrogen; SOC, soil organic carbon; NO_3_–N, nitrate nitrogen; NH4–N, ammonium nitrogen; AP, available phosphorus. *P < 0.05; **P < 0.01; and ***P < 0.001.

### The Autotrophic Bacterial Co-occurrence Networks

**FIGURE 4:**
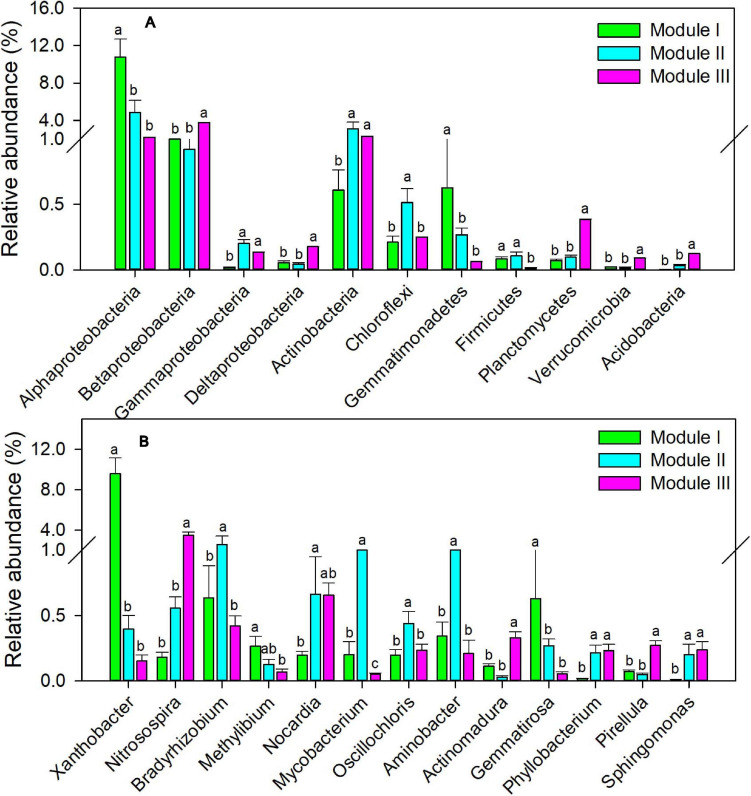
Relative abundance of different dominant modules in soil autotrophic bacterial networks at phylum **(A)** and genera **(B)** levels. Different small letters indicate the significant difference among modules at *P* < 0.05. Bars represent standard errors (*n* = 15).

To examine the co-occurrence pattern of soil *cbbL*-carrying bacterial communities in five treatments, the network was constructed from the 15 samples (Supplementary Figure 3). In total, there were 533 nodes and 4,526 links and three dominant modules (modules I, II, and III) in the autotrophic bacterial network (Supplementary Figure 3). The autotrophic bacterial network displayed more positive correlations (4,472 edges) than negative correlations (54 edges). Modules I, II, and III of the bacterial networks consisted of 122, 205, and 206 nodes, respectively. At the phylum level, the relative abundances of Alphaproteobacteria and Gemmatimonadetes were significantly greater in module I than in modules II and III; those of Betaproteobacteria, Deltaproteobacteria, Planctomycetes, Verrucomicrobia, and Acidobacteria were significantly greater in module III than in modules I and II; and those of Actinobacteria and Gammaproteobacteria were significantly greater in modules II and III than in module I (Figure 4A, *P* < 0.05). At the genus level, the relative abundances of *Xanthobacter* (Alphaproteobacteria), *Gemmatirosa* (Gemmatimonadetes), *Nitrosospira* (Betaproteobacteria), *Mycobacterium* (Actinobacteria), and *Pirellula* (Planctomycetes) followed a similar trend of individual phyla (Figure 4B, *P* < 0.05). Module I was positively correlated with NO_3_–N (*r* = 0.75, *P* < 0.01), CEE (*r* = 0.95, *P* < 0.001), and crop yield (*r* = 0.79, *P* < 0.01) (Table 3). Modules II and III were positively associated with TN (*r* = 0.59, *P* < 0.05 and *r* = 0.59, *P* < 0.05), SOC (*r* = 0.64, *P* < 0.05 and *r* = 0.78, *P* < 0.01), and TCE (*r* = 0.74, *P* < 0.01 and *r* = 0.83, *P* < 0.01) (Table 3).

### Soil Properties and Soil Autotrophic Bacterial Community Affected Crop Yield and Carbon Emission Efficiency

**FIGURE 5:**
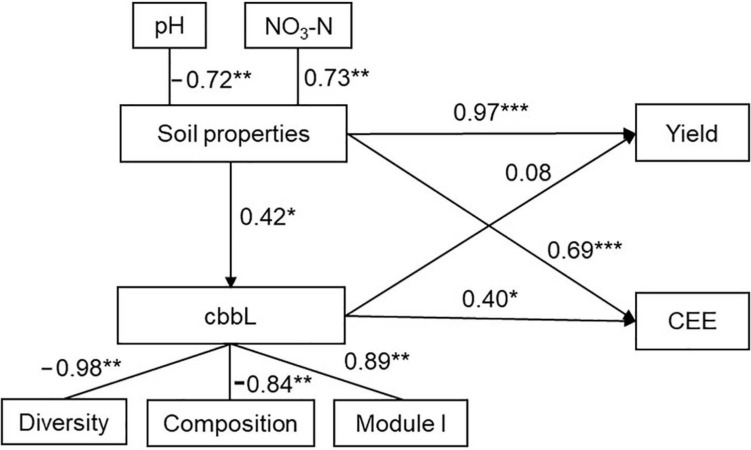
Impacts of soil properties and soil autotrophic bacterial community on crop yield and carbon emission efficiency (CEE) as estimated using structural equation modeling (SEM) analysis. Based on random forest analyses, the significant predictors were chosen to perform the SEM analysis. The latent variables inside the ellipse were used to integrate the effects of multiple conceptually related observed variables into a single-composite effect. Soil properties are represented by pH and nitrate nitrogen (NO_3_–N). Soil autotrophic bacterial community (*cbbL*) is represented by the diversity (Shannon index), composition [first principal coordinates (PCoA1)], and two modules (module II and module III) of the soil *cbbL*-carrying bacteria. *P < 0.05; **P < 0.01; ***P < 0.001.

Random forest modeling showed that soil pH (8.6% and 7.9%, *P* < 0.05) and NO_3_–N (9.4% and 8.8%, *P* < 0.01) were the two primary predictors of crop yield and CEE (Supplementary Figure 4). Module I (8.2%, *P* < 0.01; and 9.7%, *P* < 0.01) of the bacterial networks exhibited more pronounced effects on maize yield and CEE than diversity (2.5%, *P* > 0.05; and 6.5%, *P* < 0.05) and composition (1.2%, *P* > 0.05; and 6.6%, *P* < 0.05) of the autotrophic bacterial community (Supplementary Figure 4). SEM further suggested that soil properties were significantly correlated with the autotrophic bacterial community, CEE, and maize yield. The autotrophic bacterial community had strong positive relationships with CEE and crop yield according to diversity, composition, and module I of the bacterial network (Figure 5).

## Discussion

### Fertilization Treatments Affected Soil Autotrophic Bacterial Community

Based on the *cbbL* gene-carrying bacteria analysis, our results showed that organic fertilization treatments increased the diversity of soil autotrophic bacteria compared with the NA and CF treatments. Observations of large autotrophic bacterial diversity were presumably derived from changes in soil properties as affected by organic treatments. Organic fertilizer with abundant SOC and available nutrients may provide spatially adaptive living micro-habitat for the autotrophic bacterial community and thus promoted the growth of different microbial taxa and increased soil microbial diversity (Yuan et al., 2012b; Francioli et al., 2016). In addition, highest autotrophic bacterial diversity with MS suggested that maize straw are easily degradable organic materials, which provided sufficient substrate for microorganisms and increased soil microbial diversity (Yuan et al., 2012b). Soil pH has been reported as a good predictor that plays crucial roles in driving the microbial community structure and functions (Fierer and Jackson, 2006; da Jesus et al., 2009; Jiang et al., 2016). The decomposition of SOC can provide an adequate supply of nutrients and energy and significantly changed the autotrophic bacterial communities (Liao et al., 2020).

Fertilization treatments differentially modulated the autotrophic bacterial community structure with distinct shifts in bacterial diversity. The *cbbL*-carrying bacterial communities were mainly assigned to *Xanthobacter*, *Bradyrhizobium*, *Nitrosospira*, *Aminobacter*, *Mycobacterium*, *Nocardia*, *Gemmatirosa*, and *Pirellula*, etc. Most of these bacteria are previously reported as facultative autotrophs (Selesi et al., 2005; Tolli and King, 2005; Yuan et al., 2015), which play significant roles in CO_2_ fixation and degrade several carbon-containing organic compounds (Guo et al., 2015; Liao et al., 2020). However, we also found that some genera may not be autotrophs, such as the genus *Pirellula* and *Gemmatirosa* (DeBruyn et al., 2013; Glöckner et al., 2003). We further revealed that the autotrophic bacterial network contained distinct microbial modules that consisted of closely associated microbial species. The high ratio of positive edges to negative edges in the modules may suggest the ecological interactions (i.e., commensalism and mutualism) among soil autotrophic microorganisms (Zhang et al., 2018), and these associations in the microbial networks may also represent niche sharing among microorganisms (Berry and Widder, 2014). The modules in co-occurrence network generally consist of highly interconnected microorganisms and carry out various functions in maintaining ecosystem processes of nutrient exchange and resource availability (de Menezes et al., 2015; Jiang et al., 2016). Modules II and III significantly related to SOC and TN suggested that the interconnected taxa in two modules may share the same habitat preferences and common ecological niche. These modules in the bacterial network can organize a higher-order structured meta-module to reflect modules’ functional relevant interactions (Jiang et al., 2015). However, it is worth noting that caution is warranted in referring to bacterial interactions from network patterns because correlations do not necessarily represent causal relationship.

### The Mechanism of Autotrophic Bacterial Community Driving Carbon Emission Efficiency and Crop Yield

The variations in soil autotrophic bacterial community offer significant potentials for CO_2_ fixation. A study based on ^14^CO_2_ labeling has recently estimated that microbial CO_2_ assimilation processes were annually responsible for roughly 4% of the total CO_2_ fixation in terrestrial ecosystems (Yuan et al., 2012a). The present study suggested that autotrophic bacterial diversity was positively correlated with TCE and negatively correlated with CEE and plant productivity. Long-term fertilization study showed that soil autotrophic CO_2_ fixation processes were more important for the nutrient-poor environment; chemical fertilization-driving changes in soil autotrophic microbial community depressed soil CO_2_ fixation (Liao et al., 2020). In the present study, long-term organic fertilizer increased organic matter and soil autotrophic bacterial diversity but decreased CEE and crop yield as compared with chemical fertilizer treatment. These results suggested that nutrient-rich organic fertilizer treatments showed inhibitory effects on the autotrophic metabolic pathway and may consequently decrease CEE and crop yield. Theoretical and empirical evidence supported that microbial diversity plays a crucial role in predicting multiple ecological functions in terrestrial ecosystems, including nutrient cycling and climate regulation (Bardgett and Van Der Putten, 2014; Delgado-Baquerizo et al., 2016; Domeignoz-Horta et al., 2020). However, it is still a matter of debate how and to what extent soil autotrophic bacterial diversity drives microbial carbon metabolism. We speculated that the autotrophic bacterial diversity reduced soil RubisCO and CO_2_ fixation activities of bacterial populations, thereby declining CEE and crop production. High diversity suppresses microbial community function primarily due to the potential relationships among taxa in the modules of the autotrophic bacterial networks (Chen et al., 2019; Saleem et al., 2019). Highly similar microbial species shared with overlapping microhabitats (niches) may induce minimal stimulation in microbial C metabolic functioning as diversity increases (Maynard et al., 2017). In this case, reduced diversity of the autotrophic bacterial community is probably beneficial to CEE and crop yield improvement. As such, our findings highlight that soil autotrophic microorganisms may play key roles in driving carbon assimilation and contributing to soil C pool and crop productivity. However, many autotrophic microorganisms are facultative autotrophic in nature, and they have both heterotrophic and autotrophic metabolic pathways (Miltner et al., 2005). Therefore, soil autotrophic microorganisms and CO_2_ fixation only have a certain degree of correlation. Their causal relationships in the soil environment need further study. Despite that the structure and network of autotrophic bacterial community have been considered, we cannot rule out the possible impact of the *cbbL* copy numbers. Future research by stable isotope-based evidence could help gain novel insights regarding how active autotrophs interact with each other in the autotrophic bacterial community.

## Conclusion

In the present study, long-term fertilization significantly changed soil autotrophic bacterial diversity, composition, and co-occurrence network through soil pH and NO_3_–N. Soil autotrophic bacterial community had significant relationships with crop yield and CEE. Taken together, our results suggest that soil autotrophic bacterial community may drive crop productivity and CEE and mitigate greenhouse effect. Further research should continue to take advantage of pot incubation to verify the causal relationships between soil autotrophic bacterial community, crop yield, and CEE. If confirmed, we will provide novel insights into the importance of soil autotrophic bacterial community on CEE and crop yield in agricultural systems.

## Data Availability Statement

The datasets presented in this study can be found in online repositories. The names of the repository/repositories and accession number(s) can be found at: https://www.ncbi.nlm.nih.gov/bioproject/PRJNA700109.

## Author Contributions

LL, JX, and YJ designed the experiments. JW, LW, and YJ curated the literature data. JW, RZ, and YJ elaborated the data, ran the statistical analysis, and arranged the tables and figures. JW wrote the first draft of the manuscript. LL, JX, YJ, LW, and ZL critically revised the manuscript. All authors read and approved the final manuscript.

## Conflict of Interest

The authors declare that the research was conducted in the absence of any commercial or financial relationships that could be construed as a potential conflict of interest.

## Footnotes

1 http://ccb.jhu.edu/software/FLASH/

2 http://drive5.com/uparse/
