# Supplementary material for: The Impact of Fertilizer Amendments on Soil Autotrophic Bacteria and Carbon Emissions in Maize Field on the Semiarid Loess Plateau
Source: Front Microbiol. 2021 Jun 18;12:664120. doi: 10.3389/fmicb.2021.664120 (PMC8249863; doi:10.3389/fmicb.2021.664120)
Supplement: Supplementary file 1 [file Data_Sheet_1.docx]

**Supplementary materials for**

**The impact of fertilizer amendments on soil autotrophic bacteria and carbon emissions in maize field on the semiarid Loess Plateau**

**Supplementary Table 1**

**Table 1** Alpha-diversity indices of the soil autotrophic bacteria at flowering stage of maize under five treatments.

**Supplementary Figure 1–4**

**Figure 1** Diagram of the plastic film fully mulched ridge-furrow maize cropping system used in the experiment for this study at the Rainfed Agricultural Experiment Station of Gansu Agricultural University from 2012 to 2019.

**Figure 2** Relative abundance of the autotrophic bacterial genera under five treatments.

**Figure 3** Co-occurrence network of soil autotrophic bacterial communities under five treatments.

**Figure 4** Mean contribution (% of increased mean square error, MSE) of soil properties and soil *cbbL*-carrying bacterial community to yield of maize (A) and CEE (B) based on random forest modelling.

**Table 1**

Alpha-diversity indices of the soil autotrophic bacteria at flowering stage of maize under five treatments.

| Treatments ^a^ | OTUs ^b^ | Chao 1 | Shannon | Simpson |
| --- | --- | --- | --- | --- |
| NA | 5147 ± 272b ^c^ | 6962 ± 331b | 7.15 ± 0.08b | 0.0073 ± 0.0019a |
| CF | 5030 ± 398b | 6690 ± 372b | 6.91 ± 0.19b | 0.0118 ± 0.0044a |
| SC | 5608 ± 181b | 7453 ± 157b | 7.12 ± 0.14b | 0.0094 ± 0.0033a |
| SM | 5699 ± 610b | 7373 ± 809b | 7.31 ± 0.11b | 0.0056 ± 0.0012a |
| MS | 10432 ± 494a | 19364 ± 364a | 7.82 ± 0.01a | 0.0052 ± 0.0002a |

^a^ NA, no amendment; CF, chemical fertilizer; SC, 50% chemical N fertilizer plus 50% organic N fertilizer; SM, organic fertilizer; MS, maize straw.

^b^ OTUs, operational taxonomic units.

^c^ Within a column for a given dependent variable, means ± standard error followed by different letters are significantly different at *P* < 0.05.


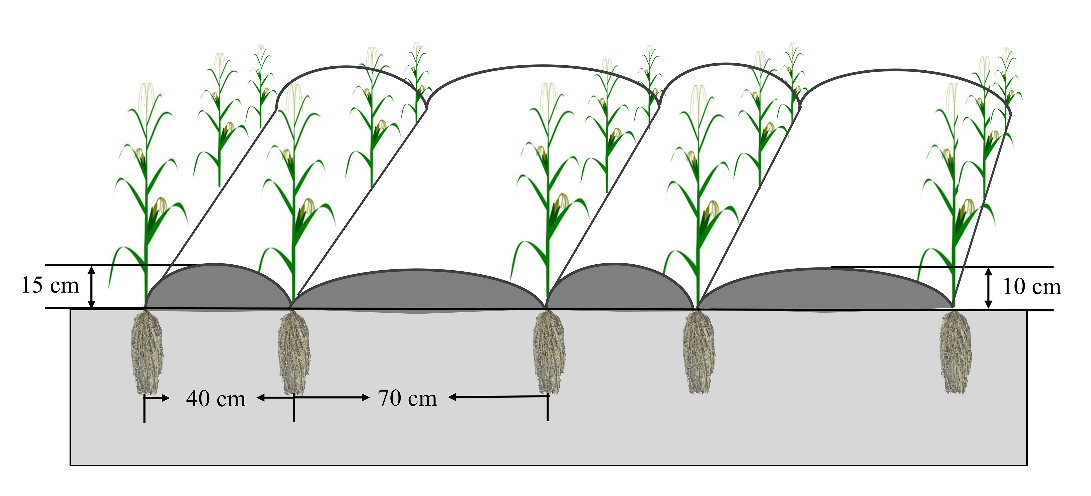


**Figure 1** Diagram of the plastic film fully mulched ridge-furrow maize cropping system used in the experiment for this study at the Rainfed Agricultural Experiment Station of Gansu Agricultural University from 2012 to 2019.


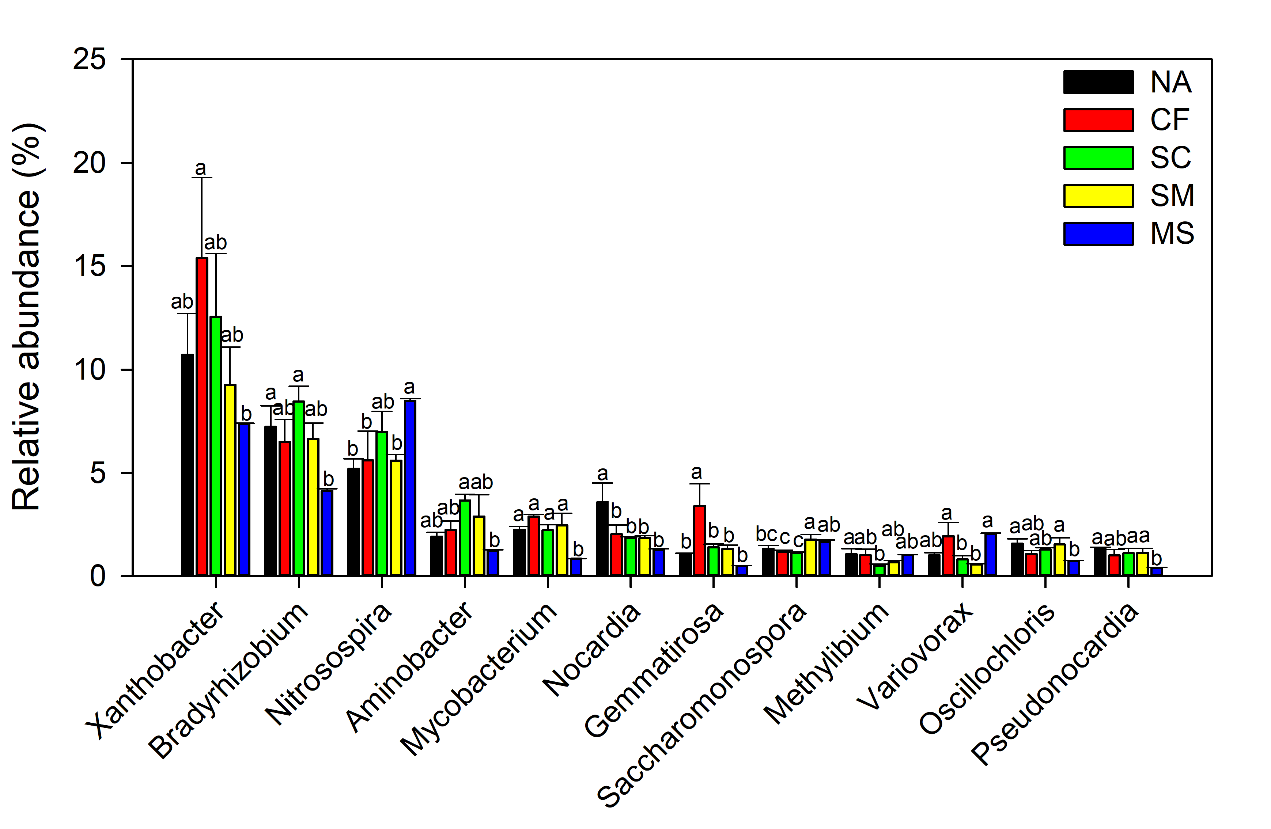


**Figure 2** Relative abundance of the autotrophic bacterial genera under five treatments. NA, no amendment; CF, chemical fertilizer; SC, 50% chemical N fertilizer plus 50% organic N fertilizer; SM, organic fertilizer; MS, maize straw. Different small letters indicate the significant difference among fertilization treatments at *P* < 0.05. Bars represent standard errors (n = 3).

**Figure 3** Co-occurrence network of soil autotrophic bacterial communities under five treatments. Networks are randomly colored by dominant modules. Modules I-III represent three clusters with closely interconnected nodes. Size of each node is proportional to the number of connections (degree), and the thickness of each connection between two nodes (edge) is proportional to the value of correlation coefficients. Blue edges indicate positive, red edges negative connections.


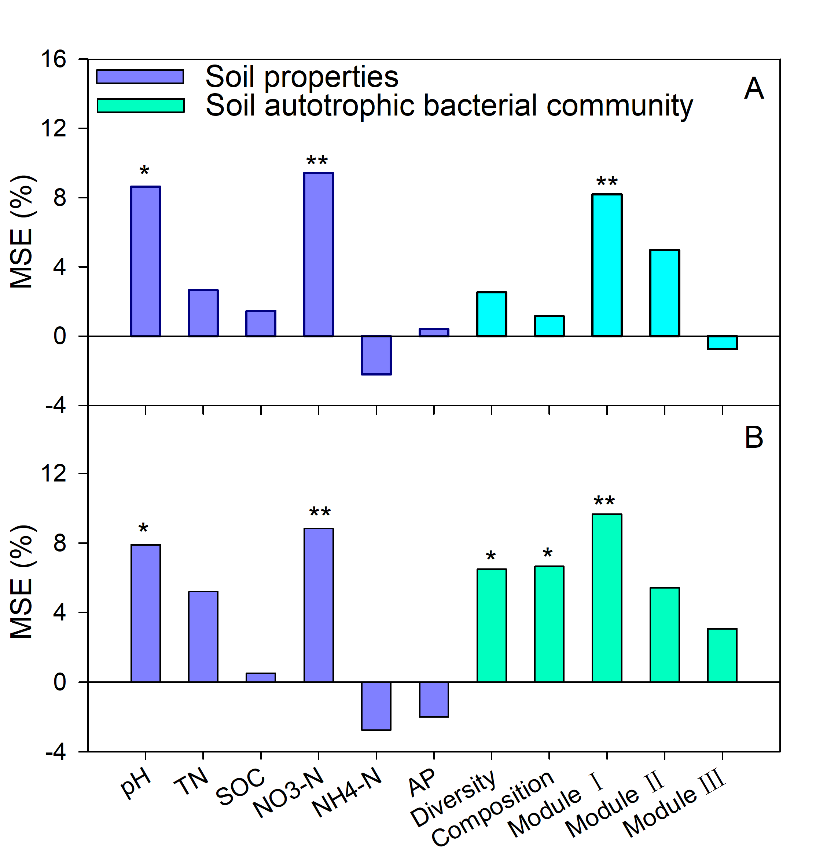


**Figure 4** Mean contribution (% of increased mean square error, MSE) of soil properties and soil *cbbL*-carrying bacterial community to yield of maize (A) and CEE (B) based on random forest modelling. Random forest modelling was performed based on 15 samples (5 treatments × 3 replicates). Soil properties include pH, total nitrogen (TN), soil organic carbon (SOC), nitrate nitrogen (NO3-N), ammonium nitrogen (NH4-N), and available phosphorus (AP). The soil *cbbL*-carrying bacterial community include diversity (Shannon index), composition (first principal coordinates, PC1), and three module eigengenes in trophic co-occurrence network. * *P* < 0.05; ** *P* < 0.01.
